# In vivo axial load-share ratio measurement using a novel hexapod system for safe external fixator removal

**DOI:** 10.1186/s12891-024-07440-y

**Published:** 2024-05-09

**Authors:** Sida Liu, Lin Lu, Tao Chen, Yanshi Liu, Dong Wei, Jun Miao, Defu Yu, Xuefei Fu

**Affiliations:** 1 School of Mechanical Engineering, Tianjin University, Tianjin, China; 2 Department of Radiotherapy, Anhui No.2 Provincial People’s Hospital, Hefei, Anhui China; 3 Department of Orthopedics, Anhui No.2 Provincial People’s Hospital, Hefei, Anhui China; 4 Department of Orthopedics, The Affiliated Hospital of Southwest Medical University, Luzhou, Sichuan China; 5 Department of Orthopedics Surgery, Tianjin Academy Traditional Chinese Medicine Affiliated Hospital, Tianjin, China; 6 Department of Spine Surgery, Tianjin Hospital, Tianjin, China

**Keywords:** Fracture healing, Callus stiffness, Axial load-share ratio, Force measurement system, Hexapod external fixator, Fixator removal

## Abstract

**Background:**

External fixation is widely used in the treatment of traumatic fractures; however, orthopedic surgeons encounter challenges in deciding the optimal time for fixator removal. The axial load-share ratio (LS) of the fixator is a quantitative index to evaluate the stiffness of callus healing. This paper introduces an innovative method for measuring the LS and assesses the method’s feasibility and efficacy. Based on a novel hexapod LS-measurement system, the proposed method is to improve the convenience and precision of measuring LS in vivo, hence facilitating the safe removal of external fixators.

**Methods:**

A novel hexapod system is introduced, including its composition, theoretical model, and method for LS measurement. We conducted a retrospective study on 82 patients with tibial fractures treated by the Taylor Spatial Frame in our hospital from September 2018 to June 2020, of which 35 took LS measurements with our novel method (Group I), and 47 were with the traditional method (Group II). The external fixator was removed when the measurement outcome (LS < 10%) was consistent with the surgeon’s diagnosis based on the clinical and radiological assessment (bone union achieved).

**Results:**

No significant difference was found in the fracture healing time (mean 25.3 weeks vs. 24.9 weeks, *P* > 0.05), frame-wearing duration (mean 25.5 weeks vs. 25.8 weeks, *P* > 0.05), or LS measurement frequency (mean 1.1 times vs. 1.2 times, *P* > 0.05). The measurement system installation time in Group I was significantly shorter compared to Group II (mean 14.8 min vs. 81.3 min, *P* < 0.001). The LS value of the first measurement in Group I was lower than that of Group II (mean 5.1% vs. 6.9%, *P* = 0.011). In Group I, the refracture rate was 0, but in Group II it was 4.3% (2/47, *P* > 0.05).

**Conclusion:**

The novel hexapod LS-measurement system and involved method demonstrated enhanced convenience and precision in measuring the LS of the external fixator in vivo. The LS measurement indicates the callus stiffness of fracture healing, and is applicable to evaluate the safety of removing the fixator. Consequently, it is highly recommended for widespread adoption in clinical practice.

## Background

External fixation plays an essential role in the treatment of high-energy fractures, bone defects, limb lengthening, and deformity correction [1, 2]. Determining the optimal time for the removal of external fixators in fracture treatment poses a significant difficulty. Prolonged utilization of the external fixator results in an uncomfortable lifestyle for patients and amplifies their psychological distress. Moreover, the fixator’s stress shielding during fracture healing will impact the bony callus formation [3, 4]. Nevertheless, premature removal of the external fixator may cause refracture [5–7]. Thus, effective evaluation of fracture healing is of crucial importance to determine the correct time of fixator removal.

At present, there is no such thing as a “Gold Standard” for determining when a fracture is fully healed. Fracture healing is generally referred to as the reconstruction of the bone’s biomechanical characteristics. Clinical and radiographic fracture healing criteria commonly applied include painless weight bearing, no discomfort at the fracture site, and “bridging or callus formation across 3 of 4 cortices” on AP and lateral x-rays [8–10]. However, it is unable of providing information regarding the biomechanical features of the bone.

Callus stiffness is the most typical biomechanical indicator of fracture healing. Currently, the in vivo evaluation of callus stiffness involves performing tests for bending [11, 12], torsion [13, 14], and axial compression [15–17]. The axial stiffness is a direct measure of the limb’s weight-bearing capacity. Aarnes et al. [18] introduced an external fixator with three load cells, and proposed an index of load-share ratio (LS) to measure the axial stiffness of the regenerate bone in vivo. The LS was calculated by dividing the force applied to the fixator by the total limb load. In their clinical study of 22 patients who underwent tibial treatment with an Ilizarov circular external fixator, the fixator was removed once the LS dropped below 10%, and none suffered refracture after the fixator removal. Their research was based on the theoretical assumption that an externally imposed load is distributed between the fixator and the regenerating bone callus, and the load carried by the bone callus is determined by its stiffness relative to the fixator. The load carried by the callus increases as it mineralizes, thus the LS decreased continuously and tended to be less than 10%.

However, certain limitations are involved in the Aarnes’ method. The interface between the fixator and the bone typically experiences complicated spatial stress, particularly notable bending stress occurring at the half-pin connection. As a result, the fixator is subjected to multi-dimensional loads. Besides, the two rings of the fixator should be parallel to each other and perpendicular to the axis of the diaphysis, which is difficult to achieve in clinical practice. Moreover, the lateral forces and bending moments acting on struts lead to inaccuracies in the force sensor.

Currently, Aarnes’ approach is still utilized for clinical assessment of LS [3, 6, 19], although it is hindered by operational difficulties and imprecise results. Thus, we have developed a novel hexapod system to overcome the discussed limitations of Aarnes’ device. This novel system was designed based on the hexapod structure like the Taylor Spatial Frame (TSF) [20, 21], which consists of six force-measuring struts connected to two rings of a ring-type external fixator. Compared with Aarnes’ devices, this hexapod structure allows a rapid exchange of the fixator’s original struts, without necessitating any alteration of the rings. Additionally, the system is a mechanism with six degrees of freedom [22], which can efficiently reduce internal stress during its installation.

This novel hexapod system was developed to measure the LS accurately and conveniently in vivo, hence improving the assessment of callus stiffness. This study aimed to provide a comprehensive description of the hexapod LS-measurement system, and evaluate its feasibility and efficacy for determining the secure time of external fixator removal.

## Methods

### Study design and patients

A retrospective analysis was conducted on patients with tibial fractures treated by TSF at Tianjin Hospital (Tianjin, China) from September 2018 to June 2020. Inclusion criteria: (1) patients with open tibial fractures or closed tibial fractures with poor soft tissue condition; (2) LS measurement was conducted when the fracture achieved bone union under clinical and radiographic examinations; (3) a minimum follow-up period of 6 months after the fixator removal. Exclusion criteria: (1) patients with bilateral tibial fractures; (2) presence of severe medical conditions; (3) pediatric fracture; (4) inability to cooperate with routine follow-up; (5) age exceeding 65 years. Eventually, a total of 82 individuals were enrolled, of which 35 were taken LS measurement with the novel method (Group I) and 47 were with the traditional method (Group II).

Authorization to utilize human subjects was acquired from the Ethics Committee of Tianjin Hospital (protocol code: 2023-MER-037), and the patients provided informed written consent. The surgical treatment and LS measurement of both groups were performed by the same team.

### Hexapod LS-measurement system

**Fig. 1:**
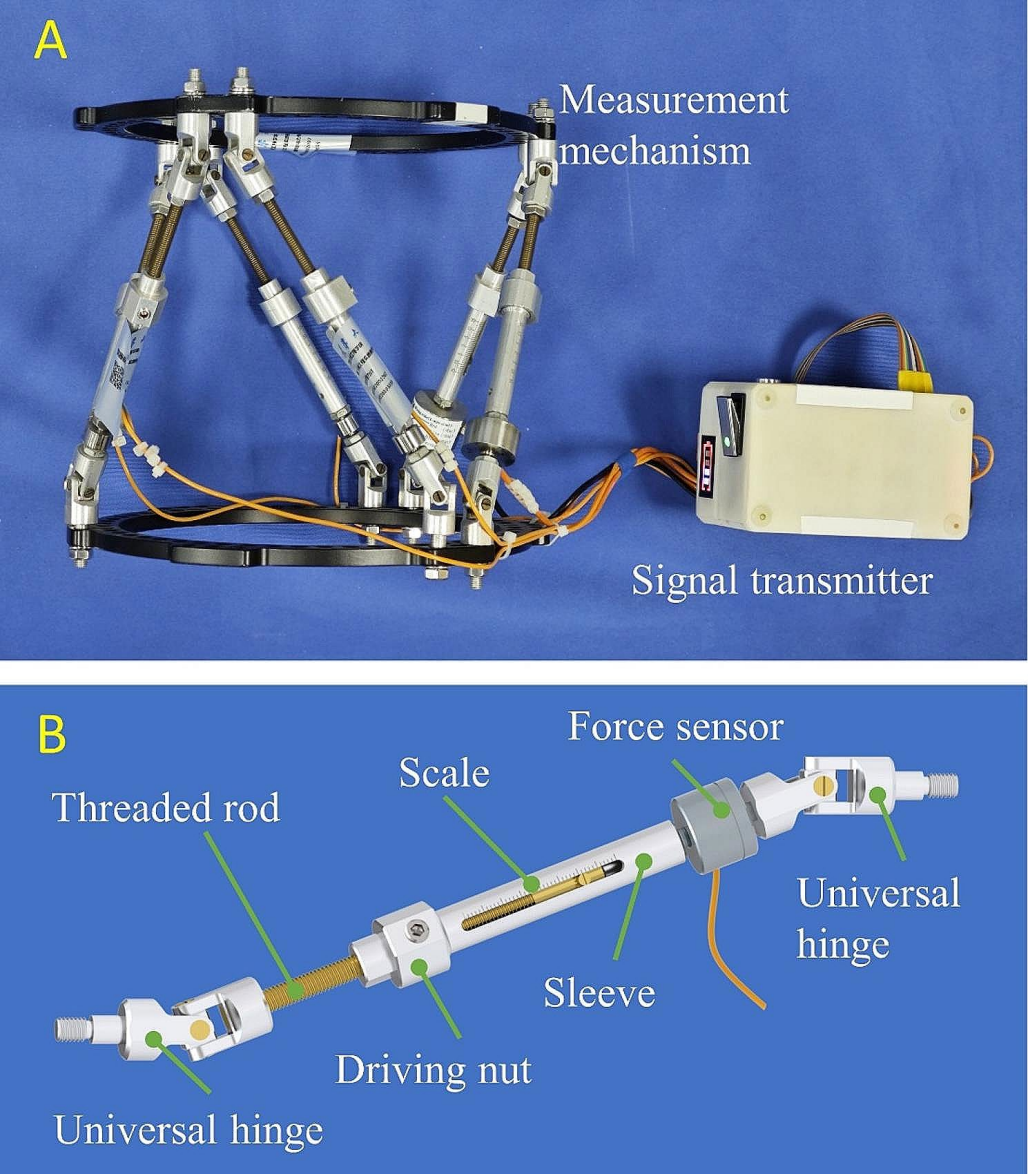
**(A)** Measurement mechanism and signal transmitter of the novel hexapod LS-measurement system. **(B)** Structure of the force-measuring strut

The novel hexapod LS-measurement system includes mechanical components, signal transmitter, and assistant computer software. The mechanical components consist of six force-measuring struts that have the same structure. These struts can be connected to the rings of different types of external fixators, constituting a hexapod measurement mechanism, as shown in Fig. 1A. The signal transmitter connects with sensors in the force-measuring struts through flexible cables. The structure of the force-measuring strut is illustrated in Fig. 1B. Each side of the strut is equipped with a universal hinge that, when coupled with the ring, creates a ball joint with three degrees of freedom. The middle section of the strut is a screw-type joint consisting of a threaded rod, a sleeve, and a driving nut. The strut’s length can be modified by twisting the driving nut, and the scale on the sleeve can determine its value. The lower section of the strut is fitted with a force sensor (DYMH-103, Dayang Sensing System Engineering Co. Ltd., China) capable of measuring an axial force in either compression or tension, with a maximum capacity of 500 N.

The signal transmitter consists of several functional parts, including the signal amplifier, analog-to-digital converter, Bluetooth communicator, and master controller. The signal transmitter converts the forces measured by six sensors into digital signals (16-bit, 6 channels), which are subsequently communicated wirelessly to a computer with the assistant software. The assistant computer software “Auto LSM” is developed using MATLAB (R2021b, MathWorks Inc., USA), which is designed to process data, conduct theoretical analysis, and eventually provide LS to surgeons.

**Fig. 2:**
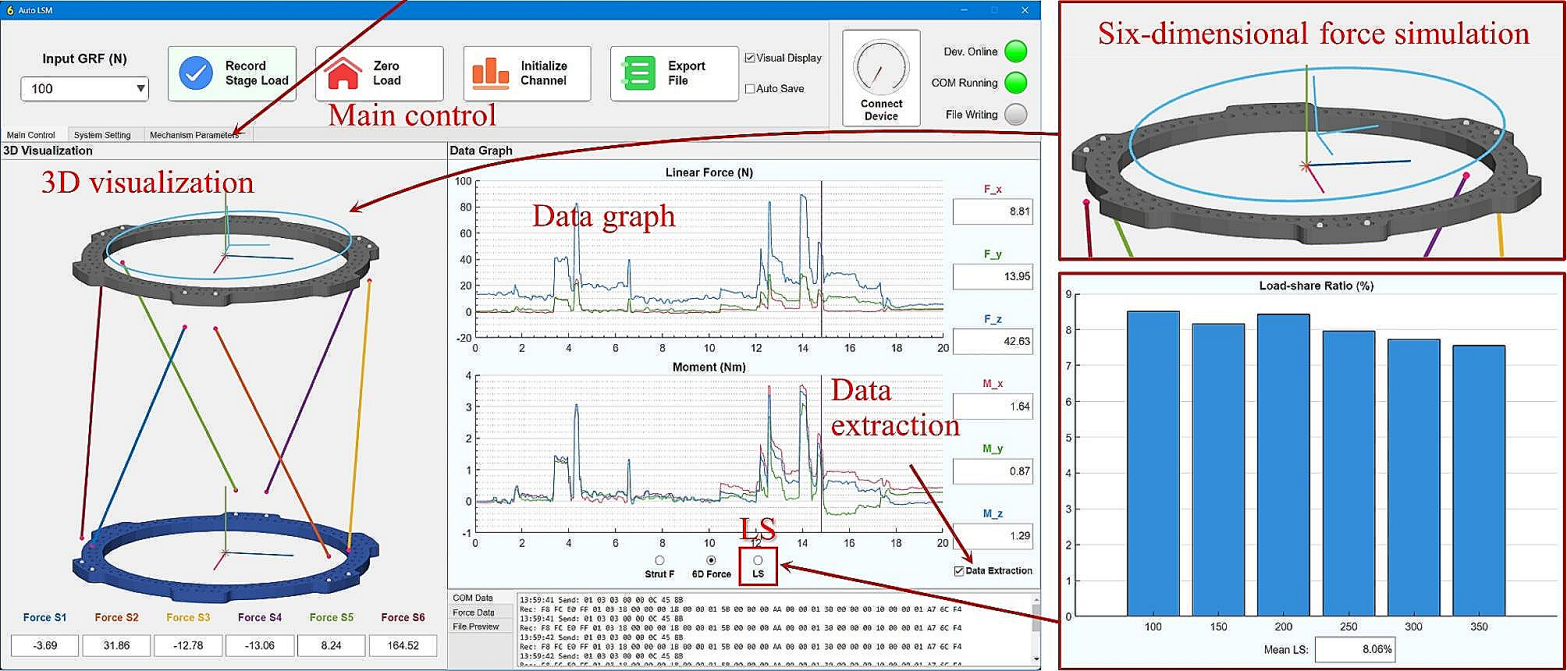
User interface of the assistant computer software named “Auto LSM”.

The Auto LSM software comprises multiple panels for assisting LS measurement and presenting results (Fig. 2). The “Main control” panel of the software provides functions controlling LS measurement, which includes inputting ground reaction force (GRF) values, recording the load of the mechanism, resetting the load value to zero, and initializing the data of each force sensor. The dimension parameters and installation parameters of the hexapod measurement mechanism is set in the “Mechanism parameters” panel. The surgeon chooses the type of the rings used, and specifies the connection position and the length of each force-measuring strut. The software then automatically calculates the configuration of the mechanism. In the “3D visualization” panel, graphical simulations of the measurement mechanism’s configuration and force are displayed. A floating coordinate frame, in conjunction with a circle, simulate the six-dimensional force exerted on the measurement mechanism. The “Data graph” panel of the software displays real-time curves of the six-dimensional force by default, while executing the “Data extraction” function can review the force information at any given moment. By pressing the “LS” button while taking measurements, a bar graph with the measured LS values and the mean LS will be displayed.

**Fig. 3:**
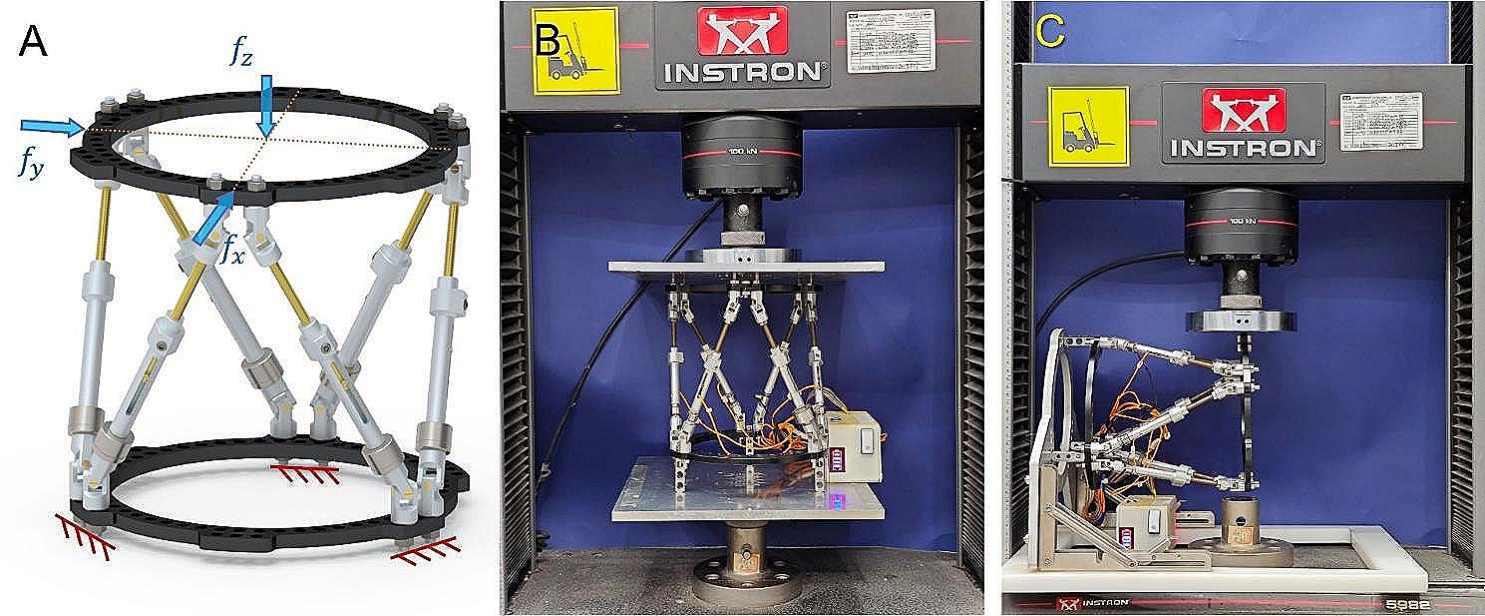
The accuracy examination of the novel LS-measurement system. **(A)** Three types of linear test loads were applied. **(B)** Loading vertically to examine the accuracy along the *z*-axis. **(C)** Loading horizontally to examine the accuracy along the *x*-axis and *y*-axis

The accuracy of the LS-measurement system has been examined by a universal testing machine (Model 5982, Instron Corp., MA, USA). Considering that the external fixator mostly experiences linear force throughout the patient’s daily functional activity, tests were conducted to examine the forces along the *x*-, *y*-, and *z*-axes. Fig. 3A depicts the position and direction of the test load being applied, while Fig. 3B and C are the views of the testing process. The test result indicated that errors along the *x*-axis and *y*-axis are both less than 0.5 N, and the error along the *z*-axis is less than 0.3 N. The LS-measurement system demonstrates a good level of precision when compared to the physiological load of the human body.

### Principles of hexapod-system-based measurement

#### Set up the frame and pose description

**Fig. 4:**
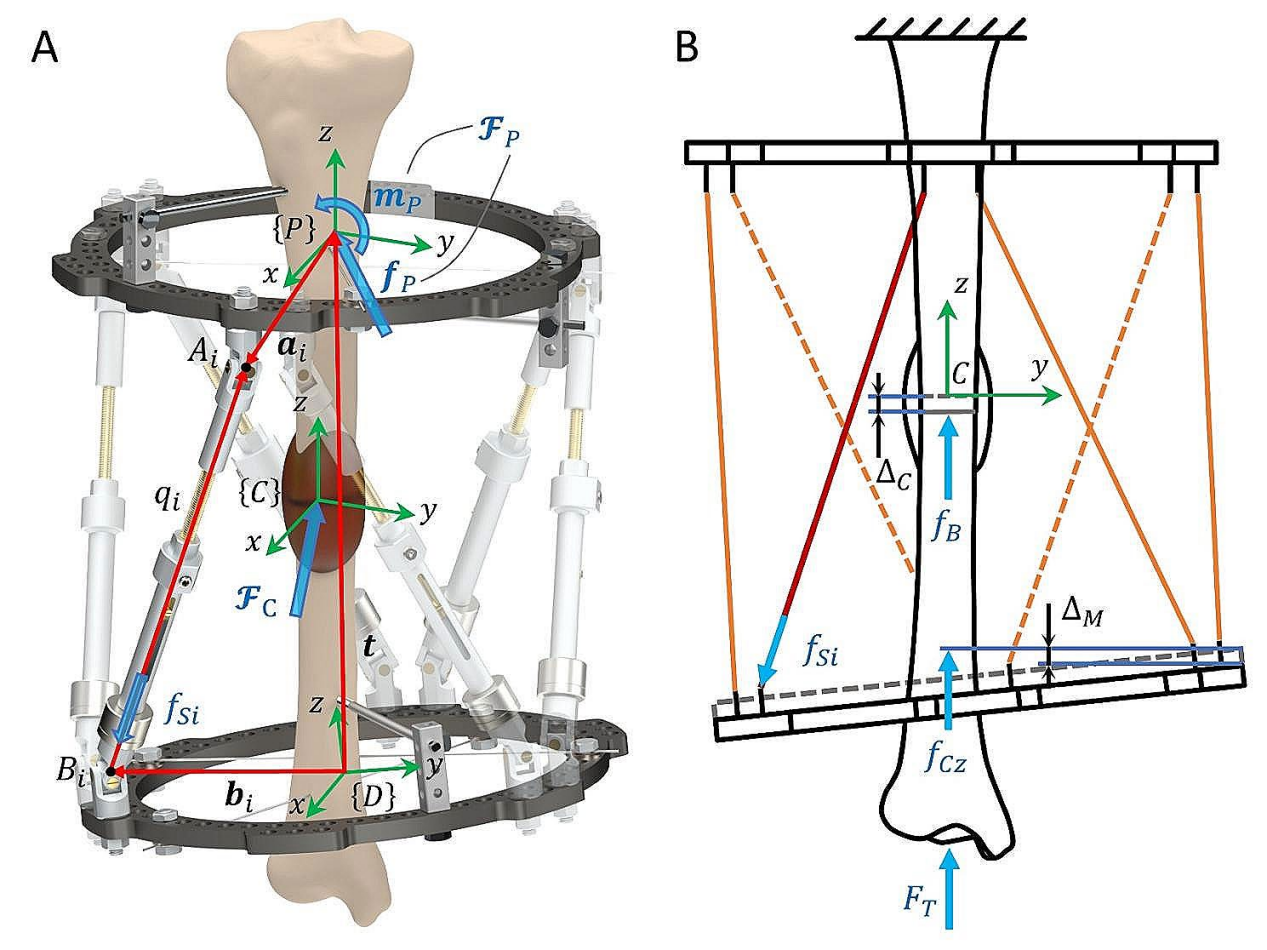
**(A)** Schematic of the bone-mechanism structure. **(B)** Axial force and deformation in the structure during LS measurement

The fractured bone and the hexapod mechanism form a bone-mechanism structure. Due to variations in patients, the lengths of struts often differ. It is necessary to first establish the description of the mechanism’s configuration. As illustrated in Fig. 4A, set cartesian coordinate frames $\left\{P\right\}$ and $\left\{D\right\}$ attached to the proximal ring and the distal ring, respectively. Simultaneously, select a frame $\left\{C\right\}$ that is connected to the fractured end (which is also the location of the callus). The bone frame $\left\{C\right\}$ is oriented with its $z$-axis aligned with the mechanical axis of the bone, while its $x$-axis and $y$-axis are directed anteriorly and laterally, respectively. The reference frame is chosen as $\left\{P\right\}$ following clinical convention, and the mechanism’s configuration can be described by the pose of the moving frame $\left\{D\right\}$, which consists of a position vector $\boldsymbol{t}$ and a rotation matrix $\boldsymbol{R}$. The rotation matrix $\boldsymbol{R}$, which has nine parameters, can be expressed as an exponential coordinate $\hat{\boldsymbol{\omega }}\theta$ with three parameters, according to the matrix exponential theory mentioned in the literature [23]. Therefore, the pose coordinate of the mechanism $\mathcal{X}$is defined as

1 $$\mathcal{X}=\left(\begin{array}{c}\boldsymbol{t}\\\hat{\boldsymbol{\omega}}\theta\end{array}\right)\in{{\mathbb{R}}^{6}}$$

#### Determine the pose of the mechanism and bone

For the $i\text{-th }(i=1,2,\cdots ,6)$ force-measuring strut, denote the center of proximal and distal universal hinge in it as point ${A}_{i}$ and ${B}_{i}$, respectively. The position vector ${\boldsymbol{a}}_{i}$ of point ${A}_{i}$ in the reference frame $\left\{P\right\}$ can be determined using the dimensional parameters of the fixator’s components. Similarly, set the position vector ${\boldsymbol{b}}_{i}$ of point ${B}_{i}$ in the moving frame $\left\{D\right\}$. The length ${q}_{i}$ of strut $i$ is defined as the distance between points ${A}_{i}$ and ${B}_{i}$, read from the sleeve’s scale.

The mechanism’s pose $\mathcal{X}$ is computed utilizing forward kinematics based on the known parameters ${q}_{i}$, ${\boldsymbol{a}}_{i}$ and ${\varvec{b}}_{i}$ specific to the patient. For the vector-loop closure of the $i\text{-th}$ strut we may write a vector equation as

2 $$\boldsymbol{t}=\boldsymbol{{a}_{i}}+{{q}_{i}}{\boldsymbol{{\hat{s}}}_{i}}-\boldsymbol{R}{\boldsymbol{b}_{i}}$$

in which the unit vector ${\hat{\boldsymbol{s}}}_{i}$ represents for the strut’s axis, and $\boldsymbol{R}{\boldsymbol{b}}_{i}$ transforms the reference of the position vector ${\boldsymbol{b}}_{i}$ to the frame $\left\{P\right\}$. Use the matrix exponential of $[\hat{\boldsymbol{\omega}}]\theta$as

3 $$\begin{matrix}   {{e}^{[\hat{\boldsymbol{\omega }}]\theta}}=\boldsymbol{I}+\sin\theta [\hat{\boldsymbol{\omega }}]+\left(1-\cos\theta \right){{[\hat{\boldsymbol{\omega}}]^{2}}}=\boldsymbol{R}  \\\end{matrix}$$

to substitute the rotation matrix $\boldsymbol{R}$ in Eq. 2, in which $\boldsymbol{I}$ represents an identity matrix and $[\hat{\boldsymbol{\omega}}]$ represents the skew-symmetric matrix of $\hat{\boldsymbol{\omega}}$. Take the norm for Eq. 2 to construct a function about$\mathcal{X}$:

4 $$\begin{matrix}   {{g}_{i}}(\mathcal{X})={{q}_{i}}-\left\|{{e}^{[\hat{\boldsymbol{\omega }}]\theta }}{\boldsymbol{b}_{i}}-{\boldsymbol{a}_{i}}+\boldsymbol{t} \right\|. \end{matrix}$$

Substitute the parameters ${q}_{i}$, ${\boldsymbol{a}}_{i}$ and ${\boldsymbol{b}}_{i}$ into the corresponding function of strut $i$, forming an equation set ${g}_{i}\left(\mathcal{X}\right)=0 \left(i=\text{1,2},\cdots ,6\right)$. The equation set has a unique solution in the workspace of the mechanism. Hence, the Levenberg-Marquardt method is used to calculate the numerical solution of the mechanism’s pose $\mathcal{X}$. Besides, the axis vector ${\hat{\boldsymbol{s}}}_{i}$ is determined by substituting $\mathcal{X}$ back to Eq. 2, which will be useful in the following.

The pose ${\mathcal{X}}_{C}$ of frame $\left\{C\right\}$ describes the bone’s spatial position relative to the reference frame $\left\{P\right\}$, which consists of a position vector ${\boldsymbol{t}}_{C}$ and a rotation matrix ${\boldsymbol{R}}_{C}$. Clinically, the values of ${\boldsymbol{t}}_{C}$ and ${\boldsymbol{R}}_{C}$ for a single patient can be acquired by measuring X-ray images and the patient’s body. Specific measurement techniques can be found in the literature [24]. The bone pose ${\mathcal{X}}_{C}$ is then derived using the matrix exponential stated above.

#### Establish the static model

Next, establish the statics model to calculate the loads applied on the hexapod measurement mechanism based on the forces acting on the six struts. The loads on the mechanism are transferred from bone pins, and can be composed into a six-dimensional force at point $P$as

5 $$\begin{array}{c}{\mathcal{F}}_{P}=\left(\begin{array}{c}{\boldsymbol{f}}_{P}\\ {\boldsymbol{m}}_{P}\end{array}\right)={\left(\begin{array}{cccccc}{f}_{Px}& {f}_{Py}& {f}_{Pz}& {m}_{Px}& {m}_{Py}& {m}_{Pz}\end{array}\right)}^{\text{T}}.\end{array}$$

Wherein, ${\boldsymbol{f}}_{P}$ represents the linear force and ${\boldsymbol{m}}_{P}$ represents the moment. The variable ${f}_{Si}$ denotes the internal force exerted on strut $i$, which can be obtained using the force sensor. Under the combined action of the six internal forces ${f}_{Si}$, the mechanism forms a static equilibrium relation with the applied loads (i.e. the six-dimensional force ${\mathcal{F}}_{P}$).

The theory of virtual work can be used to determine the loads on the mechanism. Suppose the moving frame has differential motions ${\updelta }\mathcal{X}$ due to the external force${\mathcal{F}}_{P}$, and the struts experience differential motions $$\updelta\boldsymbol{q}={\left(\begin{array}{cccc}\updelta{q}_{1}&\updelta{q}_{2}&{\cdots}& \updelta{q}_{6}\end{array}\right)}^{\text{T}}$$ of length. Based on the principle of equality between the virtual work performed by force ${\mathcal{F}}_{P}$ and by force${f}_{Si}$,

6 $$\begin{array}{c}{\mathcal{F}}_{P}^{\text{T}}\updelta\mathcal{X}={\left(\begin{array}{cccc}{f}_{S1}& {f}_{S2}& \cdots & {f}_{S6}\end{array}\right)}^{\text{T}}\updelta \boldsymbol{q}={\boldsymbol{f}}_{S}^{\text{T}}\updelta\boldsymbol{q} .\end{array}$$

A velocity equation can be obtained by taking the derivative of Eq. 2 as

7 $$\begin{array}{c}{\boldsymbol{v}}_{D}={\dot{\boldsymbol{a}}}_{i}+{\dot{q}}_{i}{\hat{\boldsymbol{s}}}_{i}+{q}_{i}{\dot{\hat{\boldsymbol{s}}}}_{i}-\dot{\boldsymbol{R}}{\boldsymbol{b}}_{i}-\boldsymbol{R}{\dot{\boldsymbol{b}}}_{i}.\end{array}$$

Notice that ${\boldsymbol{v}}_{D}$ is the linear velocity of the moving frame at point $D$. To obtain the frame’s linear velocity $\boldsymbol{v}$ at the force composition point $P$, introduce the additional velocity caused by its rotation $\boldsymbol{t}\times\boldsymbol{\omega }$ to get

8 $$\begin{array}{c}\boldsymbol{v}={\boldsymbol{v}}_{D}+\boldsymbol{t}\times\boldsymbol{\omega},\end{array}$$

in which $\boldsymbol{\omega }$ denotes the frame’s angular velocity. The derivatives ${\dot{\boldsymbol{a}}}_{i}$ and ${\dot{\boldsymbol{b}}}_{i}$ are equal to zero because the center of the universal hinge remains stationary relative to its connected ring. The derivative of the rotation matrix $\dot{\boldsymbol{R}}$ is equivalent to the cross product of the angular velocity $\boldsymbol{\omega }$ and the rotation matrix $\boldsymbol{R}$ [25], writing as $\dot{\boldsymbol{R}}=\boldsymbol{\omega }\times\boldsymbol{R}$. Combine the above analysis and take the dot product of ${\hat{\boldsymbol{s}}}_{i}$ on both sides of Eq. 7 to eliminate ${\dot{\hat{\boldsymbol{s}}}}_{i}$, we have:

9 $$\begin{array}{c}{\dot{q}}_{i}=\boldsymbol{v}\cdot {\hat{\boldsymbol{s}}}_{i}-\left(\boldsymbol{t}\times\boldsymbol{\omega}\right)\cdot {\hat{\boldsymbol{s}}}_{i}+\left(\boldsymbol{\omega }\times\boldsymbol{R}{\boldsymbol{b}}_{i}\right)\cdot{\hat{\boldsymbol{s}}}_{i}=\boldsymbol{v}\cdot {\hat{\boldsymbol{s}}}_{i}+\boldsymbol{\omega}\cdot\left(\boldsymbol{t}+\boldsymbol{R}{\boldsymbol{b}}_{i}\right)\times {\hat{\boldsymbol{s}}}_{i} .\end{array}$$

Utilize the equality of the strut axis’ moment about point $P$, expressed as $\left(\boldsymbol{t}+\boldsymbol{R}{\boldsymbol{b}}_{i}\right)\times {\hat{\boldsymbol{s}}}_{i}={\boldsymbol{a}}_{i}\times {\hat{\boldsymbol{s}}}_{i}$, and integrate the equations of struts 1–6 to rewrite Eq. 9 into matrix form:

10 $$\begin{array}{c}\dot{\boldsymbol{q}}=\left[\begin{array}{cc}{\hat{\boldsymbol{s}}}_{1}^{\text{T}}& {\left({\boldsymbol{a}}_{1}\times {\hat{\boldsymbol{s}}}_{1}\right)}^{\text{T}}\\ {\hat{\boldsymbol{s}}}_{2}^{\text{T}}& {\left({\boldsymbol{a}}_{2}\times {\hat{\boldsymbol{s}}}_{2}\right)}^{\text{T}}\\ \vdots & \vdots \\ {\hat{\boldsymbol{s}}}_{6}^{\text{T}}& {\left({\boldsymbol{a}}_{6}\times {\hat{\boldsymbol{s}}}_{6}\right)}^{\text{T}}\end{array}\right]\left(\begin{array}{c}\boldsymbol{v}\\ \boldsymbol{\omega }\end{array}\right)={\boldsymbol{J}}^{-1}\mathcal{V}.\end{array}$$

The matrix $\boldsymbol{J}$ above is the mechanism’s velocity Jacobian matrix, and $\mathcal{V}$ is the generalized velocity. Within the same time limit, the velocity $\dot{\boldsymbol{q}}$ of the driving joint and $\mathcal{V}$ of the mechanism corresponds respectively to the differential motions ${\updelta }\boldsymbol{q}$ and ${\updelta}\mathcal{X}$ in Eq. 6. Therefore, the statics model is:

11 $$\begin{array}{c}{\mathcal{F}}_{P}=\left[\begin{array}{cccc}{\hat{\boldsymbol{s}}}_{1}& {\hat{\boldsymbol{s}}}_{2}& \cdots & {\hat{\boldsymbol{s}}}_{6}\\ {\boldsymbol{a}}_{1}\times {\hat{\boldsymbol{s}}}_{1}& {\boldsymbol{a}}_{2}\times {\hat{\boldsymbol{s}}}_{2}& \cdots & {\boldsymbol{a}}_{6}\times {\hat{\boldsymbol{s}}}_{6}\end{array}\right]{\boldsymbol{f}}_{S}=\boldsymbol{G}{\boldsymbol{f}}_{S}.\end{array}$$

To calculate the specific force mapping matrix $\boldsymbol{G}$ of each patient, determine the position vector ${\boldsymbol{a}}_{i}$ and the axis vector ${\hat{\boldsymbol{s}}}_{i}$ as mentioned above. By substituting the force of sensors ${\boldsymbol{f}}_{S}$ into Eq. 11, the six-dimensional force ${\mathcal{F}}_{P}$ of the mechanism is obtained.

The tibia is mainly subjected to pressure along its mechanical axis under daily physiologic load. Therefore, transform the six-dimensional force ${\mathcal{F}}_{P}$ acting on the mechanism to the point $C$ in frame $\left\{C\right\}$, and determine its projection along the $z$-axis to evaluate bone healing. By applying the coordinate transformation equation for six-dimensional forces, we can determine the load of the mechanism at the callus location:

12 $$\begin{array}{c}{\mathcal{F}}_{C}=\left[\begin{array}{cc}{\boldsymbol{R}}_{C}^{\text{T}}& 0\\ {-\boldsymbol{R}}_{C}^{\text{T}}[{\boldsymbol{t}}_{C}]&{\boldsymbol{R}}_{C}^{\text{T}}\end{array}\right]{\mathcal{F}}_{P}.\end{array}$$

Wherein, ${\boldsymbol{R}}_{C}^{\text{T}}$ represents the transpose of the rotation matrix ${\boldsymbol{R}}_{C}$ and $[{\boldsymbol{t}}_{C}]$ stands for the skew-symmetric matrix of the position vector ${\boldsymbol{t}}_{C}$. Then, take the $z$-component of the six-dimensional force ${\mathcal{F}}_{C}$ to get the force along the tibia’s mechanical axis:

13 $$\begin{array}{c}{f}_{Cz}=\left(\begin{array}{cccccc}0& 0& 1& 0& 0& 0\end{array}\right){\mathcal{F}}_{C} .\end{array}$$

#### Calculate the LS

Eventually, calculate the LS to assess the callus stiffness. The LS is defined as the ratio between the load applied to the measurement mechanism and the entire bone-mechanism structure [18]. The force and deformation along the diaphysis axis during LS measurement are illustrated in Fig. 4B. The entire axial load exerted on the structure ${F}_{T}$ can be measured by a weight scale. The axial force transferred to the mechanism ${f}_{Cz}$ is the linear force along the *z*-axis of the six-dimensional force ${\mathcal{F}}_{C}$. Hence the LS value can be calculated as

14 $$\text{LS}=\frac{{{f}_{Cz}}}{{{F}_{T}}}$$

During the static measurement of the LS, the load of bone-mechanism structure ${F}_{T}$ can be considered composed of the axial force transferred through the bone ${f}_{B}$ and that through the mechanism ${f}_{Cz}$, expressed as ${F}_{T}={f}_{B}+{f}_{Cz}$. Further, integrate the relationship between force and deformation to obtain:

15 $$\begin{array}{c}\text{LS}=\frac{{f}_{Cz}}{{f}_{B}+{f}_{Cz}}=\frac{{k}_{M}{{\Delta }}_{M}}{{k}_{B}{{\Delta }}_{B}+{k}_{M}{{\Delta }}_{M}}\approx \frac{{k}_{M}}{{k}_{B}+{k}_{M}} .\end{array}$$

Wherein, ${k}_{M}$, ${{\Delta }}_{M}$, ${k}_{B}$ and ${{\Delta }}_{B}$ are the stiffness of the mechanism, deformation of the mechanism, stiffness of the callus, and deformation of the callus, respectively. The amount of deformation generated by the callus is approximately the same as that generated by the mechanism, ${{\Delta }}_{B}\approx {{\Delta }}_{M}$, thus obtaining the right side of Eq. 13. The stiffness of the callus ${k}_{B}$ increases with its consolidation, therefore, the LS provides a quantified indication of fracture healing.

### Assessment before LS measurement

During the patient’s postoperative follow-up, surgeons confirmed the tibial fracture had healed according to clinical and radiographic criteria, which included painless weight bearing, no discomfort at the fracture site, and “bridging or callus formation across 3 of 4 cortices” on AP and lateral X-rays. Once the regenerate bone callus met the above criteria, the surgeon conducted the following LS measurement.

Firstly, the original fixator configuration including struts’ lengths and their installation positions was recorded. Then, the hinges that connect the struts and the rings were loosened. The patient was advised to engage in weight-bearing exercises such as walking and stair climbing for roughly 30 min. If the fracture site exhibited pressing pain, longitudinal tapping pain, or aberrant movement, it was necessary to reconnect the hinge and restore the original state of the external fixator. The next LS measurement would be conducted after a 4-week interval. If not, proceed to the subsequent LS measurement steps.

### Hexapod-system-based LS measurement

**Fig. 5:**
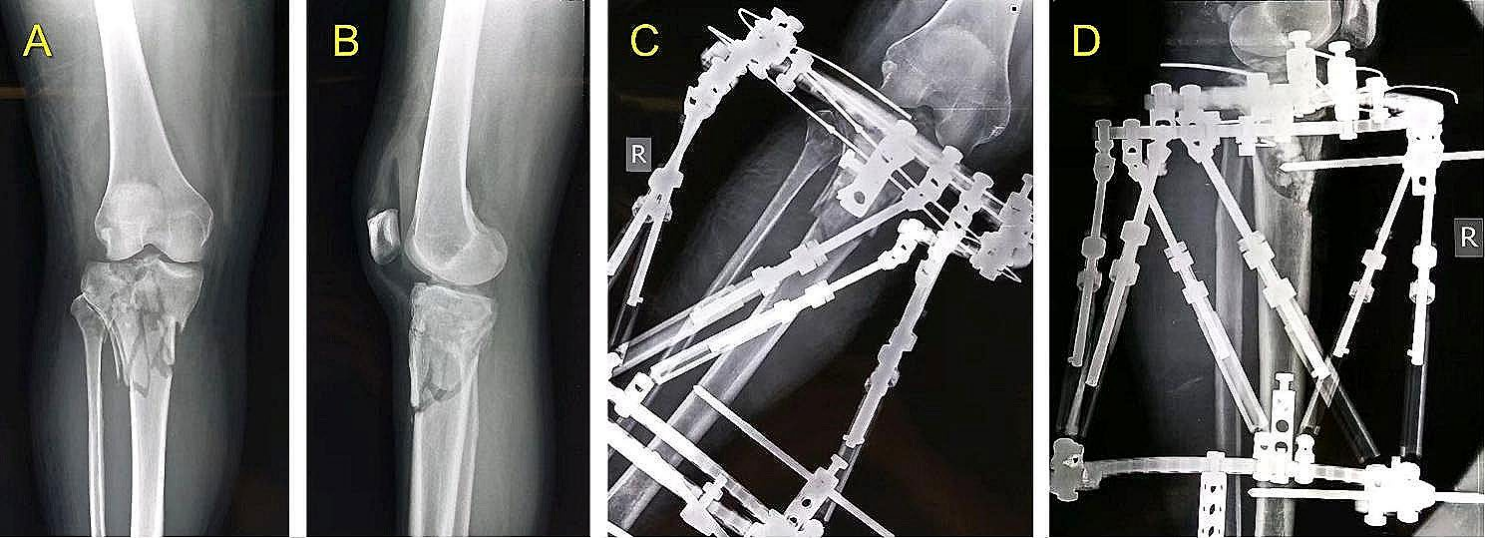
A 47-year-old female who suffered tibial fracture in right leg and treated by the TSF external fixator. **(A, B)** Immediate AP and lateral views of X-rays after injury. **(C, D)** Immediate AP and lateral views of X-rays after surgery

**Fig. 6:**
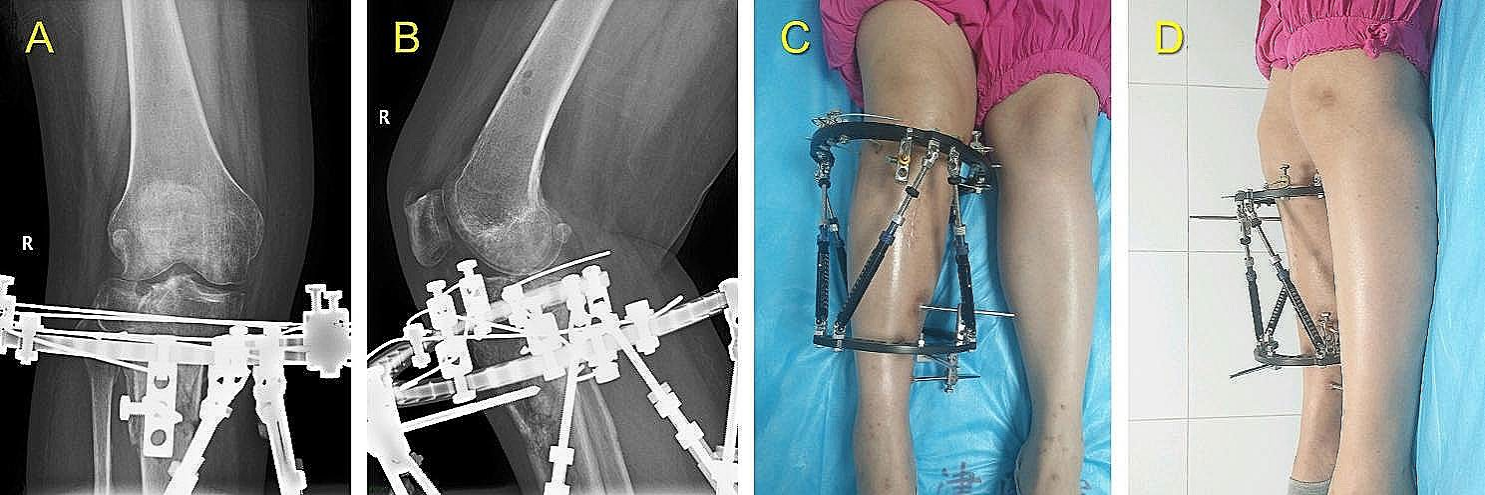
The last follow-up images of the same patient shown in the Fig. 5 before removing the TSF. **(A, B)** AP and lateral views of X-rays 24 weeks after surgery. **(C, D)** Clinical AP and lateral views 24 weeks after surgery

**Fig. 7:**
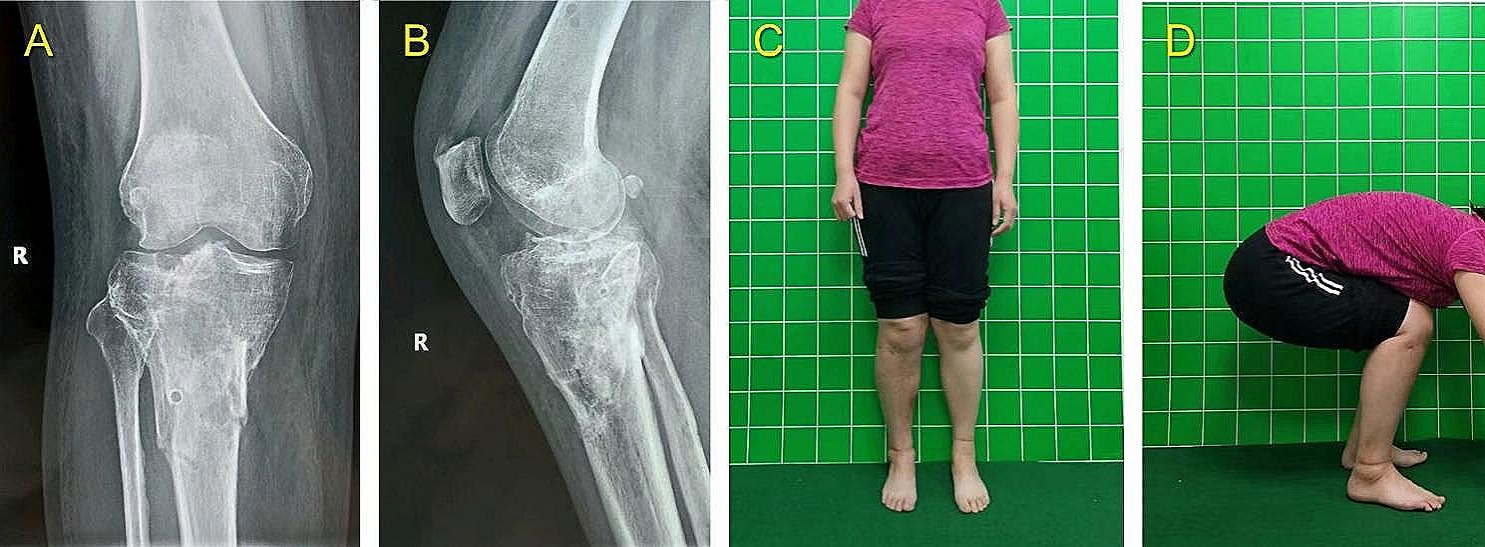
The follow-up images of the same patient shown in the Fig. 5 after removing the TSF. **(A, B)** AP and lateral views of X-rays 4 weeks after TSF removal. **(C, D)** Clinical AP and lateral views 4 weeks after TSF removal

**Fig. 8:**
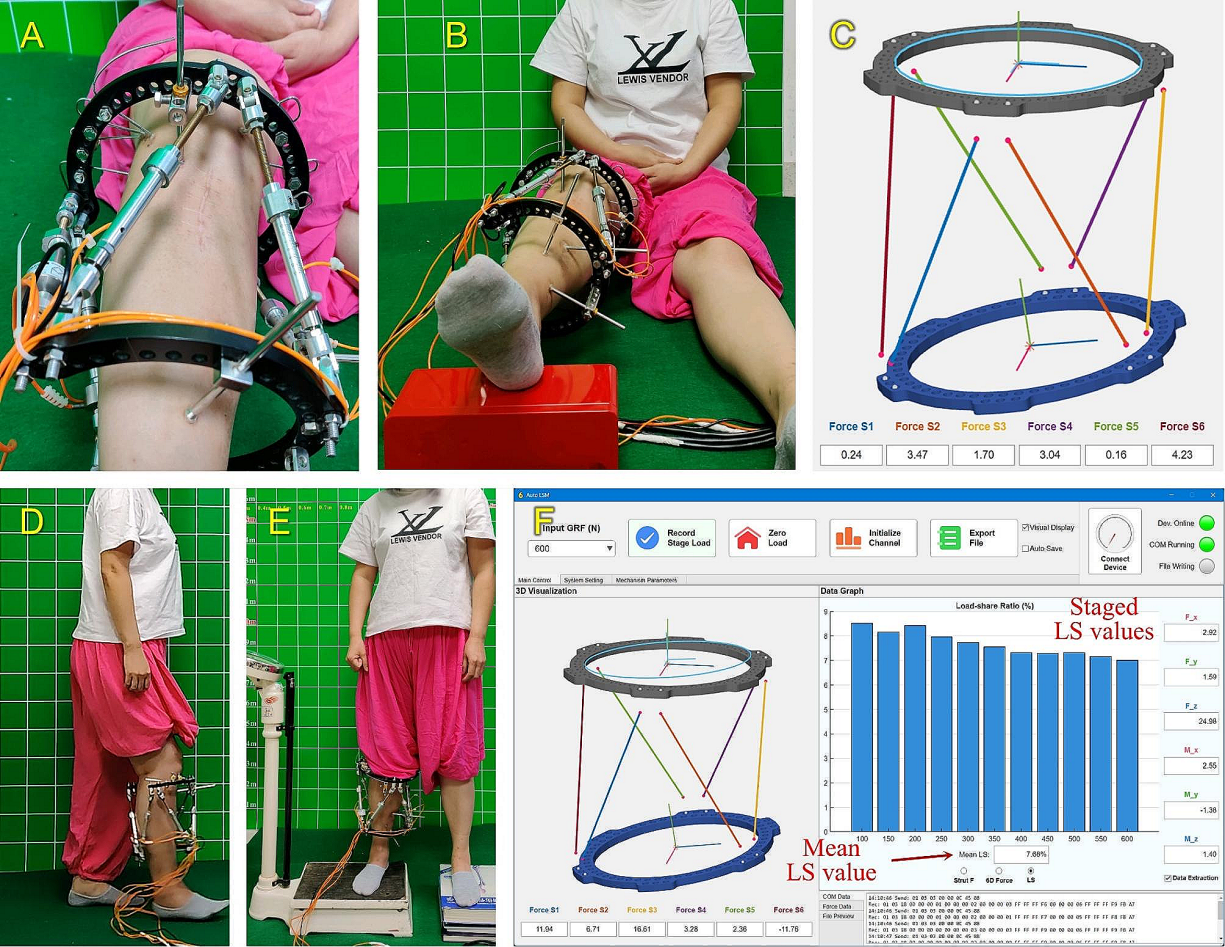
Photographs and software screenshots of the LS measurement procedure using the novel hexapod system. **(A)** Install the hexapod measurement mechanism by exchanging the struts. **(B)** Lift the affected limb horizontally to adjust the strut force to the initial value. **(C)** Graphical simulation of the installed mechanism in the Auto LSM software **(D)** Take adaptive activities soon after the system installation. **(E)** Conduct LS measurement. **(F)** LS outcomes in the software

In Group I, LS measurement was performed based on the novel hexapod system. The method consists of the following five steps. A typical case is shown in Figs. 5, 6 and 7, and 8.

#### Initialize the system

Firstly, the signal transmitter was activated to establish communication with the computer. Then, the doctor ran the Auto LSM software and executed the “Connect Device” function to verify if the software’s force signals react to actual changes in strut forces. Next, the force-measuring struts were horizontally positioned to minimize experiencing external forces. At last, the “Initialize Channel” function was executed by the system to initialize the sensor signal.

#### Install the hexapod measurement mechanism

The original struts were removed and exchanged with the force-measuring struts. To prevent additional stress on the bone pins, the two rings should remain steady. The hexapod measurement mechanism had been successfully installed (Fig. 8A). The affected limb was horizontally lifted, and then the force-measuring struts’ lengths were adjusted to create a minimal compression force ranging from 0 to 5 N (Fig. 8B and C).

#### System function test

The lengths and installation positions of force-measuring struts were recorded and inputted into the software. The software then automatically performed theoretical calculations. The surgeon verified the congruity between the software’s graphical simulation and the mechanism’s actual configuration (Fig. 8C). Simultaneously, the patient took adaptive activities for approximately five minutes (Fig. 8D).

#### LS measurement

A weight scale and a platform were placed at the same height. The patient stood with the affected limb on the weight scale and the healthy limb on the platform (Fig. 8E). The GRF (N) of the affected limb was converted from the scale reading (kg). The procedures were as follows: First, the affected limb was raised slightly to achieve the GRF of zero, and the “Zero Load” function was executed. Then, the GRF test value was set as the starting value of 100 N, with staged increments of 50 N. Especially, the final test value corresponded to the maximum load-bearing capability of the affected limb. Next, the patient loaded the affected limb referring to the staged GRF test value, and the surgeon then input this value into the software and executed the “Record Stage Load” function. Finally, the software measured and calculated the LS value. The patient underwent three rounds of LS measurement.

#### Complete the measurement

After each measurement round, a series of staged LS values was obtained, which was displayed automatically in the software interface (Fig. 8F). The final LS result was determined by calculating the mean of LS values across all stages in three rounds of measurement.

### Traditional LS measurement

**Fig. 9:**
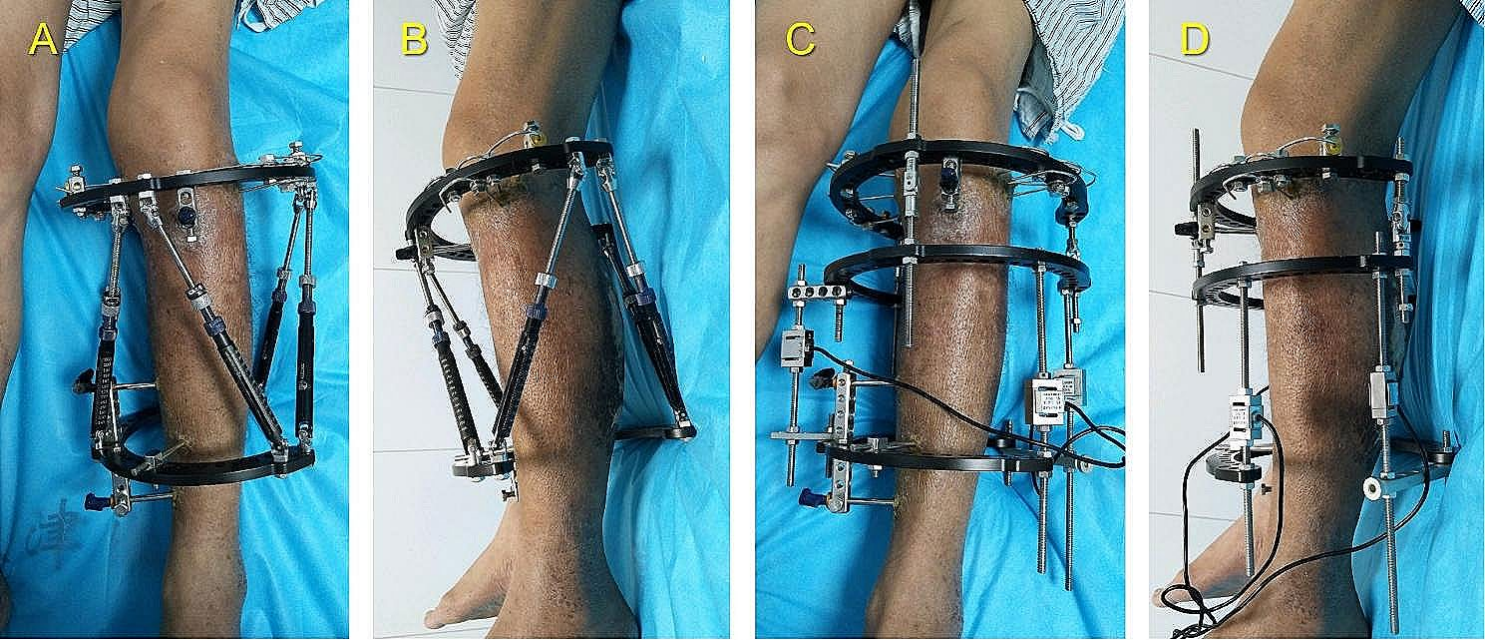
A 55-year-old male who suffered tibial fracture in left leg and treated by TSF.

In Group II, the LS value was measured using the traditional device and method, as detailed in our earlier study [6]. The original struts were removed while the integrity of rings and pins was preserved. If the two rings were aligned in parallel, traditional force-measuring struts could be installed vertically between them; if not, the measurement mechanism would require supplementary components. A case shown in Fig. 9 was taken as an example: a 2/3 ring was connected to the proximal ring through several hinges and studs, a parallel relationship was established between the 2/3 ring and the distal ring. Afterward, three traditional force-measuring struts were installed vertically.

**(A, B)** Clinical AP and lateral views 16 weeks after surgery. **(C, D)** Installation of the traditional LS-measurement device with several supplementary components.

### Clinical treatments after measurement

The ultimate determination to remove the TSF, both for patients in Group I and Group II, depends on two factors: firstly, the bone callus satisfies the clinical and radiological standards, and secondly, the LS value is below 10%.

If the LS result was less than 10%, the patient’s callus was considered to achieve sufficient stiffness, and subsequently, the external fixator was removed. If the LS result exceeded 10%, the initial external fixation condition was restored, and functional exercise was continued. A clinical visit and LS measurement were taken after a 4-week period. The external fixator was removed until the LS value was below 10%.

### Statistical analysis

The following parameters were recorded in both groups: general data, fracture healing time, frame-wearing duration, LS measurement frequency, measurement system installation time, LS value of the first measurement, and refracture rate.

The statistical study was conducted using SPSS software (22.0, IBM Corp., USA). The analysis of continuous variables was performed using independent-sample *t*-tests. The results were presented as the mean, standard deviation, and range of the observations. The count variables were examined by the Chi-square or Fisher’s test. A difference was considered statistically significant if the *P*-value was less than 0.05.

## Results

The demographic statistics of the two groups are presented in Table 1, and there was no statistically significant difference (*P* > 0.05). In Group I, there were 8 cases of proximal tibial fractures (4 of type A, 2 of type B, and 2 of type C). There were 24 cases of tibial shaft fracture (16 of type A and 8 of type B). There were 3 cases of distal tibial fractures (2 of type A and 1 of type B). In Group II, there were 10 cases of proximal tibial fractures (6 of type A, 3 of type B, and 1 of type C). There were 33 cases of tibial shaft fracture (20 of type A, 11 of type B, and 2 of type C). There were 4 cases of distal tibial fracture, all of which were type A. Within Group I, there were a total of 9 cases of open fractures and 26 cases of closed fractures. Within Group II, there were 13 cases of open fractures and 24 cases of closed fractures. As for the Gustilo’s classification, there were 4 cases of type I and 5 cases of type II in Group I; there were 3 cases of type I, 8 cases of type II and 2 cases of type III in Group II. No significant statistics difference was found in fracture type (AO, open/closed and Gustilo’s classification).

**Table 1:** Details of patients in the two groups

	Group I	Group II	Statistical value	*P*-value
Mean age in years (range)	45.8 ± 13.6 (19 to 65)	42.6 ± 11.6 (21 to 62)	1.150	0.254
Gender (male : female)	26 : 9	32 : 15	0.372	0.542
Fracture type (AO classification)				
41 A : 41B : 41 C	4 : 2: 2	6 : 3 : 1	0.720	0.698
42 A : 42B : 42 C	16 : 8 : 0	20 : 11 : 2	1.535	0.464
43 A : 43B : 43 C	2 : 1 : 0	4 : 0 : 0	1.556	0.212
Open/closed fracture				
Open	9	13	0.039	0.844
Closed	26	34		
Gustilo’s classification				
Type I	4	3	2.180	0.336
Type II	5	8		
Type III	0	2		

The clinical results are presented in Table 2. The mean fracture healing time was 25.3 ± 5.4 weeks in Group I and 24.9 ± 4.6 weeks in Group II (*P* > 0.05). In Group I, the mean frame-wearing duration was 25.5 ± 5.2 weeks, compared to 25.8 ± 3.9 weeks in Group II (*P* > 0.05). The mean measurement system installation time in Group I (14.8 ± 4.6 min) was shorter than that in Group II (81.3 ± 39.3 min), and the difference was statistically significant (*P* < 0.001).

**Table 2:** Comparison of clinical outcomes between the two groups

Variable	Group I	Group II	Statistical value	*P*-value
Fracture healing time (week)	25.3 ± 5.4	24.9 ± 4.6	0.349	0.728
Frame-wearing duration (week)	25.5 ± 5.2	25.8 ± 3.9	−0.321	0.749
LS measurement frequency (time)	1.1 ± 0.2	1.2 ± 0.5	−1.483	0.142
Measurement system installation time (minute)	14.8 ± 4.6	81.3 ± 39.3	−9.959	< 0.001
LS value of the first measurement (percentage)	5.1 ± 2.7	6.9 ± 3.4	−2.605	0.011
Refracture rate (percentage)	0	4.3	1.527	0.505

The mean LS value of the first measurement in Group I was 5.1 ± 2.7% and 6.9 ± 3.4% in Group II, and there was a statistical difference (*P* = 0.011). In Group I, 33 patients underwent fixator removal after the first LS measurement, while 2 patients (the first LS was 11.5% and 12.6%, respectively) had their fixators removed after the second measurement. In Group II, 40 patients had their fixators removed after the first measurement. Following the second measurement, 5 patients (the first LS was 10.8%, 11.5%, 11.9%, 12.6%, and 13.5%, respectively) underwent fixator removal. Further, 2 patients (the first LS was 14.7% and 15.2%, respectively) in Group II had their fixators removed after the third test.

None of the 35 patients in Group I suffered refracture after fixator removal. Conversely, two patients in Group II suffered refracture, resulting in a refracture rate of 4.3%. Normal walking was the mechanism responsible for one patient who suffered refracture 5 weeks after fixator removal. And for another patient, walking up and down stairs led to refracture 6 weeks after fixator removal. After conducting a thorough clinical and radiographic examination, the first LS measurements met the specified LS criteria (lower than 10%), with values of 8.5% and 9.1% respectively. No statistical significance was found in the refracture rate between the two groups (*P* > 0.05).

## Discussion

Our results showed that the novel hexapod system can greatly shorten the installation time compared with traditional Aarnes’ devices. In Group I, the mean installation time was 14.8 ± 4.6 min, significantly shorter than the 81.3 ± 39.3 min in Group II. This can be explained by the fact that six struts can be rapidly connected to the rings, without necessitating any alteration of the rings, which can facilitate the installation procedure and save time in clinical practice. In our study, the refracture rate was considered a vital indicator to evaluate the novel hexapod system’s feasibility and efficacy for determining the secure time of external fixator removal. The results of our study indicated that the refracture rate in Group I was 0%, but in Group II it was 4.3% (2/47), which was due to the more precise LS outcome obtained by the novel hexapod system.

External fixation is a well-established technique in fracture and deformity treatments with several benefits, including the preservation of soft tissues, limited surgical intervention, and early mobilization [26, 27]. The prolonged use of external fixators can cause significant discomfort and necessitate ongoing nursing, so patients aspire to remove the external fixator as early as possible. Besides, Sumner et al. [28] pointed out that the stress shielding of the fixator diminishes the necessary mechanical stimulation at the fracture site. Thus, removing the fixator later will impact the process of fracture healing. Nevertheless, if the mechanical stiffness of the callus is inadequate, the early fixator removal can result in bone malunion or even refracture. Simpson et al. [7] documented 180 instances of distraction osteosynthesis with external fixator, with a refracture ratio of 9.4% after fixator removal. According to Krettek C et al. [29], the refracture ratio after fixator removal was 6%. Liu et al. [6] reported 52 cases of tibial fractures treated by external fixation, in which 4 patients suffered refracture following the fixator removal, with a refracture ratio of 7.7%. Therefore, timing fixator removal is an essential decision for a satisfactory outcome.

Current methods for assessing fracture healing rely on subjective clinical and radiological evaluation [30]. Corrales et al. [31] reviewed 123 studies about radiographic fracture healing, involving 11 different criteria used to define fracture union, however, the reliability of the radiographic assessment of fracture healing was reported only in two studies. Furthermore, Anand et al. [32] assessed the intraobserver and interobserver reliability of plain radiographs when applying an external fixator, and found that the agreement between the involved surgeons was less than 50%. Thus, evaluating fracture healing objectively and precisely is of great significance in clinical practice.

Callus stiffness can be effectively measured, especially in fractures treated by external fixation [33, 34]. The present researches on callus stiffness include axial, bending, and torsional stiffness. Measuring axial stiffness provides a more direct approach to assessing the load-bearing capacity of the affected limb. Several studies [3, 6, 18, 19] have demonstrated that LS measurement is an effective method for evaluating the axial stiffness of the callus in vivo. This method works upon the principle that an externally applied compressive load distributes between the fixator and the bone. As the fracture heals and the callus stiffness increases, the fixator bears less load, resulting in a reduced value of LS.

Aarnes’ device utilized in the traditional LS measurement consists of three struts equipped with force sensors. It has certain installation requirements: (1) The two rings are perpendicular to the diaphysis axis and parallel to one another. (2) Each strut is coaxial with the force sensor contained within it, and is parallel to the diaphysis axis. (3) The rings and the interconnected struts are at right angles to each other. However, it is difficult to fully meet these requirements in clinical practice for several factors. Firstly, the relative position of the rings and bone is subjectively controlled by the surgeon during operative installation. Secondly, after the fracture reduction using the fixator, it is common for the two rings to become axial misalignment. Thirdly, the soft tissue surrounding the fracture commonly undergoes stress relaxation during the healing process, potentially changing the force distribution of the external fixator. To solve the above problems, our surgeons and mechanical engineers cooperated to develop a novel hexapod LS-measurement system. The system’s automatic computation and visualization of LS, along with its easy installation and operation, significantly reduces the effort required by surgeons who lack engineering expertise.

The hexapod mechanism of the novel system was designed based on the Gough-Stewart platform, with universal hinges and a driving joint in each force-measuring strut [22]. This structural feature allows a rapid exchange of the fixator’s original struts, without necessitating any alteration of the rings. Hence, the novel system can be readily adjusted to work with several ring-type fixators, such as Ilizarov, TSF, and TrueLok-Hex. The study in this paper collected patients who received fracture treatment with the TSF. The novel system formed the same configuration as the original fixator (Figs. 6 and 8) and successfully conducted LS measurement in vivo. In contrast, the traditional method necessitated supplementary connecting components to meet installation requirements, leading to a complex operation procedure. The mean measurement system installation time in Group I was 14.8 ± 4.6 min, significantly shorter than the 81.3 ± 39.3 min in Group II. This emphasizes the convenience of the novel system in clinical application. In addition, the novel system is also compatible with monolateral and hybrid fixators, simply by transforming them into a two-ring structure.

The device used in the traditional method shares the same structure with the Ilizarov fixator. However, this hyperstatic structure is prone to generating internal stress, resulting in additional load on the callus. Furthermore, the mechanics theory employed by the traditional method is a simplified one-dimensional model, which considers the sum of the struts’ force as the load applied to the fixator. The load that passes from the bone to the fixator is a complex spatial force, particularly the transverse forces and bending moments that are created by half-pins [35, 36]. Non-negligible transverse force and bending moments were also observed during the six degree-of-freedom force measurement in our study. Generally, the *x*-axis force and *y*-axis force accounted for 10–40% of the *z*-axis force, with one case even reaching 80%.

In Group II, two patients unfortunately suffered refracture after the fixator removal. They met the specified criteria (LS < 10%) at the first measurement (LS was 8.5% and 9.1%, respectively), and their duration of frame-wearing was close to the mean of Group II (24 and 22 weeks, respectively). It was determined that the external fixator could be removed without extending the duration of wearing the frame. Furthermore, among the seven patients in Group II who extended the duration of wearing the frame (LS > 10% in the first measurement), no refracture occurred after the fixator removal. The issue of inadequate bone repair observed in Group II, specifically when the LS is less than 10%, further showed the inaccuracy in LS measurement using the traditional method. All patients in Group I accomplished bone repair, without any instances of refracture. This study demonstrated that the novel LS measurement method offers more accurate guidance for the removal of external fixators.

In Group I, the LS > 10% in the first measurement occurred in two cases, while in Group II it was in seven cases. In addition, the LS value of the first measurement in Group I (5.1% ± 2.7) was significantly smaller than that in Group II (6.9% ± 3.4). For most cases in Group II, supplementary components were attached, leading to extra forces exerted on the sensors. This accounts for the greater LS value found in Group II. To ensure the safety of fixator removal for patients with LS > 10%, we prolonged the duration of frame wearing and conducted later LS measurement. This explains the higher LS measurement frequency and longer frame-wearing duration in Group II.

This study has several limitations: The hexapod LS-measurement system can measure dynamic six-dimensional force. Yet when determining the static LS value, we only consider the force exerted along the *z*-axis, which is inadequate for assessing the dynamic spatial load bearing experienced by patients throughout their daily activities. The study lacked a control group for patients whose fixator removal was only determined by clinical criteria. Furthermore, all participants received treatment with the TSF without any other types of external fixators.

In the future, our team will carry out further research, which may include adding a plantar pressure measurement device to analyze dynamic LS changes, expanding the scope of clinical trials (such as Bastiani and Ilizarov), and advancing the system’s structure and functionality to enable long-term wearable measurement. Besides, we will focus on developing a particular modular TSF with the characteristics of a hexapod external fixator, providing better stabilization and superior accuracy in correcting fractures. In addition, the uniqueness of modular TSF is that it employs load cells directly attached to the six struts, which can monitor the load carried by TSF in real time, further calculating the LS of regenerate bone callus. Real-time LS feedback on bone callus can effectively predict the risk of refracture and correctly guide the dynamization of TSF, promoting the union of regenerate bone callus.

## Conclusion

Before the removal of the external fixator, a more comprehensive and objective assessment of fracture healing can be obtained by considering both clinical criteria and the axial load-sharing ratio (LS) of the fixator. This paper introduced a novel hexapod system for measuring LS in vivo, which was demonstrated to be both convenient and precise in clinical application. The external fixator can be safely removed when the LS value, as measured by this system, is below 10%.

## Data Availability

The datasets analysed during the current study are available from the corresponding author on reasonable request.

## Declarations

The studies involving humans were approved by the Ethics Committee of Tianjin Hospital (protocol code: 2023-MER-037). The studies were conducted in accordance with the local legislation and institutional requirements. All patients in this study had provided their written informed consent to participate in this study.

## Abbreviations

LS: Axial load-share ratio
TSF: Taylor Spatial Frame
GRF: Ground reaction force
