# Correction: In vivo axial load-share ratio measurement using a novel hexapod system for safe external fixator removal

**DOI:** 10.1186/s12891-024-07535-6

**Published:** 2024-06-06

**Authors:** Sida Liu, Lin Lu, Tao Chen, Yanshi Liu, Dong Wei, Jun Miao, Defu Yu, Xuefei Fu

**Affiliations:** 1 School of Mechanical Engineering, Tianjin University, Tianjin, China; 2 Department of Radiotherapy, Anhui No. 2 Provincial People’s Hospital, Hefei, Anhui China; 3 Department of Orthopedics, Anhui No. 2 Provincial People’s Hospital, Hefei, Anhui China; 4 Department of Orthopedics, The Affiliated Hospital of Southwest Medical University, Luzhou, Sichuan China; 5 Department of Orthopedics Surgery, Tianjin Academy Traditional Chinese Medicine Affiliated Hospital, Tianjin, China; 6 Department of Spine Surgery, Tianjin Hospital, Tianjin, China

Correction: *BMC Musculoskelet Disord***25**, 353 (2024)

10.1186/s12891-024-07440-y

Following publication of the original article [1], the authors corrected an error in the affiliations. Sida Liu, Lin Lu and Tao Chen were incorrectly assigned to three of same affiliations [Affiliations 1,2,3] in the published article. However, Sida Liu belongs to affiliation 1 only, Lin Lu belongs to affiliation 2 and Tao Chen belongs to affiliation 3.

The affiliations of the mentioned authors have been updated above and the original article [1] has been corrected.
